# Construction of a two-parameter empirical model of left ventricle wall motion using cardiac tagged magnetic resonance imaging data

**DOI:** 10.1186/1475-925X-11-79

**Published:** 2012-10-24

**Authors:** Jack J Shi, Mohammed Alenezy, Irina V Smirnova, Mehmet Bilgen

**Affiliations:** 1 Physics and Astronomy, University of Kansas, Lawrence, KS, 66045, USA; 2 Physical Therapy and Rehabilitation Science, University of Kansas Medical Center, Kansas City, KS, 66160, USA; 3 Radiology Department, Faculty of Medicine, Erciyes University, Kayseri, 38039, Turkey

**Keywords:** Cardiac modeling, Myocardial deformation, Left ventricle wall motion, Tagged MRI

## Abstract

**Background:**

A one-parameter model was previously proposed to characterize the short axis motion of the LV wall at the mid-ventricle level. The single parameter of this model was associated with the radial contraction of myocardium, but more comprehensive model was needed to account for the rotation at the apex and base levels. The current study developed such model and demonstrated its merits and limitations with examples.

**Materials and methods:**

The hearts of five healthy individuals were visualized using cardiac tagged magnetic resonance imaging (tMRI) covering the contraction and relaxation phases. Based on the characteristics of the overall dynamics of the LV wall, its motion was represented by a combination of two components - radial and rotational. Each component was represented by a transformation matrix with a time-dependent variable α or β.

Image preprocessing step and model fitting algorithm were described and applied to estimate the temporal profiles of α and β within a cardiac cycle at the apex, mid-ventricle and base levels. During this process, the tagged lines of the acquired images served as landmark reference for comparing against the model prediction of the motion. Qualitative and quantitative analyses were performed for testing the performance of the model and thus its validation.

**Results:**

The α and β estimates exhibited similarities in values and temporal trends once they were scaled by the radius of the epicardium (*r*_*epi*_)and plotted against the time scaled by the period of the cardiac cycle (*T*_*cardiac*_) of each heart measured during the data acquisition. α/*r*_*epi*_ peaked at about Δ*t*/*T*_*cardiac*_=0.4 and with values 0.34, 0.4 and 0.3 for the apex, mid-ventricle and base level, respectively. *β*/*r*_*epi*_ similarly maximized in amplitude at about Δ*t*/*T*_*cardiac*_=0.4, but read 0.2 for the apex and - 0.08 for the base level. The difference indicated that the apex twisted more than the base.

**Conclusion:**

It is feasible to empirically model the spatial and temporal evolution of the LV wall motion using a two-parameter formulation in conjunction with tMRI-based visualization of the LV wall in the transverse planes of the apex, mid-ventricle and base. In healthy hearts, the analytical model will potentially allow deriving biomechanical entities, such as strain, strain rate or torsion, which are typically used as diagnostic, prognostic or predictive markers of cardiovascular diseases including diabetes.

## Background

Research in biomechanical modeling of left ventricle (LV) aims to determine sensitive and specific entities that can serve as clinical biomarkers for evaluating cardiac function or diagnosing an abnormality. With this in mind, rigorous computational models have been developed for mimicking the LV wall motion and determining global and regional parameters for assessing cardiac performance [1-6]. Any measured deviation in a model parameter of interest from its normative value is considered as an indication of an abnormality and offers key diagnostics for detecting dysfunction associated with heart disease.

In general, the LV wall motion of a healthy heart in short-axis view is a complex event involving actions of radial and rotational components during both systolic contraction and diastolic relaxation phases of the cardiac cycle [7]. However, the LV wall at mid-ventricle moves mostly in radial direction, meaning it contracts isotropically with negligible amount of rotation. Based on these unique characteristics, a simple one-parameter model was developed earlier to mathematically represent the motion field at the mid-ventricle [8]. The current study was under taken to increase the capacity of this empirical model further to account for the twist-type motions of the apex and base. Specifically, a new parameter was introduced into the previous model. As before, the new proposed model with now two parameters was tested if it could sufficiently accomplish this task. The validation work was performed with the cardiac data acquired from healthy human hearts using tagged magnetic resonance imaging (tMRI) modality [9].

In the following, we first describe the assumptions and considerations that led to the construction of the two-parameter model. Next, we summarize the tMRI data acquisition protocol for visualizing the heart under clinical situation and introduce image preprocessing and registration algorithms for fitting the model to the data. Then, we give details of the construction of the model and demonstrate its capabilities with examples. Finally, we discuss the results and state our conclusions.

### Two-parameter model of the LV wall motion

#### Justification of the model

The short axis of the left ventricle is defined as the 2D polar transverse plane perpendicular to its long axis. When the contraction of a healthy heart is visualized in this view during systole, the LV wall appears rotating with respect to its long axis while simultaneously shortening radially [7]. The rotation profile appears non-uniform. When the heart is viewed from head-to-toe direction, both apex and base rotate counter-clockwise at the beginning of the systole, but shortly after the rotation changes direction to become clockwise at the apex while it remains counter-clockwise at the base. The twist action induces torsion between the apex and base [10]. Mathematical modeling of the LV wall motion has been the focus of the past and current research efforts. Lack of detailed information on the material properties and structural organization of the myocardial tissue makes it difficult to develop a comprehensive model simulating the complete motion profile of the LV wall accurately. Under a set of specific assumptions and considerations, however, reliable and repeatable models can be constructed to represent certain aspects of the LV wall motion. For example, from dynamic tMRIs, noticing that the rotation of the LV wall is negligible at the mid-ventricle level led to the formulation of a one-parameter model [8]. The current study extends this concept further and expresses the rotation as a superposition to the radial motion by introducing a second parameter into the previous one-parameter model. The necessary and sufficient conditions for the formulation of the two-parameter LV motion model can be summarized as:

1) LV shape is governed by a cylindrically symmetric geometry along its long-axis.

2) Origin of the coordinates is positioned at the center of the LV wall in the short-axis view.

3) Beginning of the systolic contraction corresponds to the end-diastolic phase denoted by time *t*=0. At this moment, the LV wall appears at its most dilated state and experiences no deformation or torsion.

4) Vectors **r**=(*x,y,t*=0) and **r**^′^=(*x*^′^,y^′^*t*) denote Cartesian coordinates of the myocardial tissue in the LV wall in the short-axis plane before (at the end-diastole) and after going through the radial motion and/or rotation during either the contraction or relaxation phases of the LV. The difference **r**^′^**r** denotes displacement. We note that this time-dependent vectorial representation is consistent with Lagrangian description of the motion [11].

5) Magnitudes $r=\left|r\right|={\left(x^{2}+y^{2}\right)}^{1/2}$ and $r'=\left|r'\right|={\left(x{'}^{2}+y{'}^{2}\right)}^{1/2}$ are distances to the origin before and after the motion of the myocardial tissue, respectively.

6) The new spatial coordinates of a moving myocardial tissue are written in terms of its position at the end-diastole. This requires breaking up the motion into its radial and rotational components. Each component is independently represented by a combination of an isotropic radial transformation and a non-uniform rotational transformation in two-dimensional space, respectively.

7) The shortening of the LV wall along its long axis during the contraction or relaxation produces negligible amount of out-of-plane motion.

#### Formulation of the isotropic radial transformation

Under the conditions stated above, the in-plane isotropic radial motion from the coordinate $r=\left[\begin{array}{c} x\\ y \end{array}\right]$ at time *t*=0 to $r'=\left[\begin{array}{c} x^{'}\\ y^{'} \end{array}\right]$ at time *t* was related by a transformation **T**[8]

(1) r'=Tr

where $T=\left[\begin{array}{cc} 1-\frac{\alpha^{2}\left(t\right)}{\left(x^{2}+y^{2}\right)} & 0\\ 0 & 1-\frac{\alpha^{2}\left(t\right)}{\left(x^{2}+y^{2}\right)} \end{array}\right]$.

α(*t*) is a time-dependent parameter. The minus sign in the diagonal elements of **T** indicates contraction. Rearranging Eq. (1) yields radial tissue displacement |**r** ^′^ − **r**| = *α*^2^/|**r**| that is inversely proportional to the distance to the origin. 1/*r*-dependence in the displacement reflects that the motion is more significant near the inner boundary (endocardium) than the outer boundary (epicardium) of the LV wall. Larger α yields larger displacement. Similarly, |**r** ' − **r**|/|**r**| = *α*^2^/|**r**|^2^ indicates that the relative displacement is inversely proportional to the square of the distance to the origin.

#### Formulation of the non-uniform rotational transformation

A pure rotation by an angle Θ is expressed by the transformation

(2) r'=Rr=

where $r=\left[\begin{array}{c} x\\ y \end{array}\right]$, $r'=\left[\begin{array}{c} x^{'}\\ y^{'} \end{array}\right]$ and $R=\left[\begin{array}{cc} \cos\Theta & -\sin\Theta \\ \sin\Theta & \cos\Theta \end{array}\right]$ .

However, close examination of the in vivo images of heart indicates that the myocardium goes through a lesser degree of rotation near the endocardium than the epicardium [12]. Our empirical analysis of the images in polar coordinates suggested that the radially non-uniform behavior of the rotation can be absorbed into the formulation by the substitution Θ=β(*t*)/|**r**|. In this notation, a positive (or negative) β corresponds to a clockwise (or counterclockwise) rotation. Larger β corresponds to an increase in rotation, but β=0 represents no rotation as in the case experienced by the myocardium of the mid-ventricle.

#### Forward transformation

Combining Eqs. (1) and (2) yields the timewise forward transformation **r**^′^=**RTr** or $\left[\begin{array}{c} x^{'}\\ y^{'} \end{array}\right]=\left[\begin{array}{l} \left(1-\frac{\alpha^{2}\left(t\right)}{r^{2}}\right)\left\{x\;\cos\frac{\beta \left(t\right)}{r}-\;y\;\sin\frac{\beta \left(t\right)}{r}\right\}\\ \left(1-\frac{\alpha^{2}\left(t\right)}{r^{2}}\right)\left\{x\;\cos\frac{\beta \left(t\right)}{r}+\;y\;\sin\frac{\beta \left(t\right)}{r}\right\} \end{array}\right]$ where

(3) $$r={\left(x^{2}+y^{2}\right)}^{1/2}$$

The time-dependent variables α and β together constitute the two parameters of the model whose values at time *t* to be estimated by following the algorithm described below.

It should be noted that in order to avoid nonlinear entanglement of the cascaded transformations in Eq. (3), Eq. (1) is first applied orderly before Eq. (2) since 1/*r* is invariant under rotation but the angle Θ changes with the application of the radial transformation in Eq. (2). This ordering reduces the complexity of the computational process in estimating the parameters α and β to describe the underlying real motion of the LV wall. The inverse transformation associated with the time-reversal operation was given in Appendix.

In practice, to successfully characterize the spatial and temporal profile of the LV wall motion or the other features of the motion dynamics, the analytical model in Eq. (3) needs to be validated by comparing the model based motion predictions against the real motion of the LV wall during the different phases of the cardiac cycle - while contracting in the systolic phase or relaxing in the diastolic phase. The steps involved in the validation process are described next.

## Materials and methods

### tMRI data acquisition

**Figure 1:**
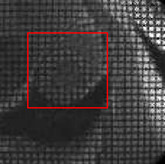
**Tagged MRI of a human heart.** Magnetization tag lines are the dark lines forming a uniform two dimensional mesh in the image. The square window identified with the red colored edges and is used for cropping the image at the center of the LV.

Cardiac tagged MRI (tMRI) modality was employed for visualizing the regional dynamics of the beating hearts in healthy humans (n=5; 3 males and 2 females) [9]. The procedures for the scans performed in this work were approved by the institutional committee governing the human studies. The tagged image contains a mesh of dark lines for labeling the material coordinates of the underlying myocardial tissue. Figure 1 shows examples of tagged images acquired from short-axis view of a human heart while undergoing contraction. As seen in the images, the tag lines warp with the motion of the LV wall and thereby provide critical information regarding the regional contraction and viability of the myocardial tissue.

**Figure 2:**
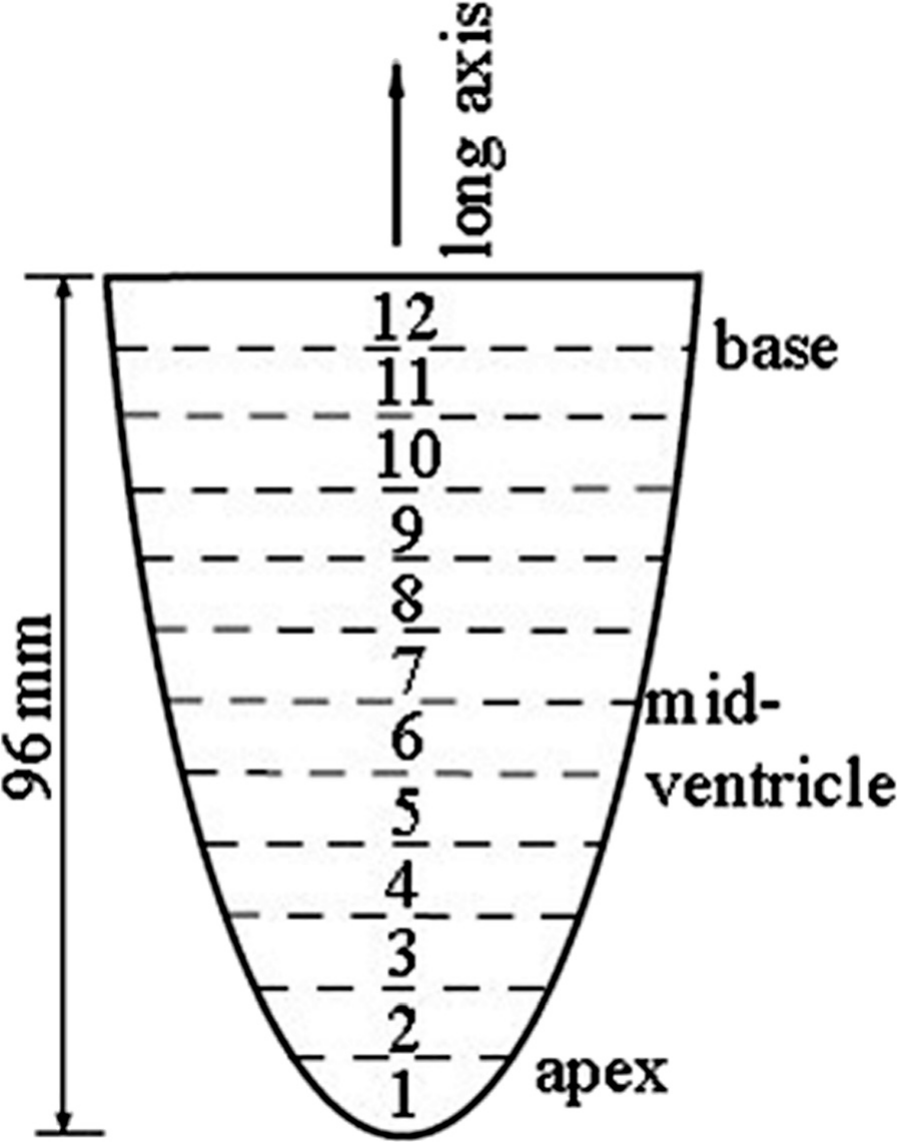
**Selection of slices for viewing the LV from its short**-**axis.** Slice numbering starts from the apex and increased towards the base. Slice thickness is adjusted so that twelve slices cover the LV fully.

tMRI data were acquired using a 3 T clinical scanner (Model Signa HDxt, GE Healthcare, Inc., Milwaukee, Wisconsin) with HD cardiac coil. The acquisition was gated by ECG and respiratory signals and the imaging parameters were: TR/TE = 4.8/2.2 ms, flip angle = 10 ms, bandwidth = 498 Hz, number of averages = 1, field of view = 350×400 mm^2^, and image matrix = 372×512 pixels yielding an in-plane resolution of 0.941×0.781 mm^2^. The tag lines were separated by 7.0 mm and the tag width was 1.5 mm. The LV was divided into 12 slices along its long axis, as shown in Figure 2. The thickness of each slice was 8 mm with no gap in between . The slices fully covered the apex, mid-ventricle and base levels and the LV was viewed from the head-to-toe direction. The full period of the cardiac cycle was divided into 11 equal-spaced time intervals and the first frame corresponded to the end-diastole when the LV was fully dilated.

### Preprocessing of the tMRI data

An algorithm was given earlier for the processing of the acquired tMRI data sets prior to performing the motion analysis on the LV wall [8]. But, the current study applied a slightly different preprocessing approach to reduce the level of complexity. This was achieved by normalizing the variables in the formulation by the key parameters describing the shape and size of the LV wall. The preprocessing step involved locating the center (long-axis) of the LV wall and its epicardial and endocardial radii in every 1^st^ end-diastolic image from different slices. For example, to accomplish this task at the slice corresponding to the mid-ventricle level, a series of stroboscopic images were first selected from the underlying slice location. The 1^st^ image frame in the series was displayed to depict the end-diastolic LV on the computer screen. The LV wall on this image appeared at its largest cross sectional dimension compared to the remaining images in the series. Then, a circle was aligned with the epicardium of the LV wall. The center and radius of the circle were taken as the center and epicardial wall radius (*r*_*epi*_) of the LV at the end-diastole. *r*_*endo*_ was similarly estimated from a smaller circle by aligning its circumference with endocardium. These parameters for the LV wall in the other slices at the apex and base levels were also measured similarly by repeating the above process on the corresponding image frames.

### Validation of the model

**Figure 3:**
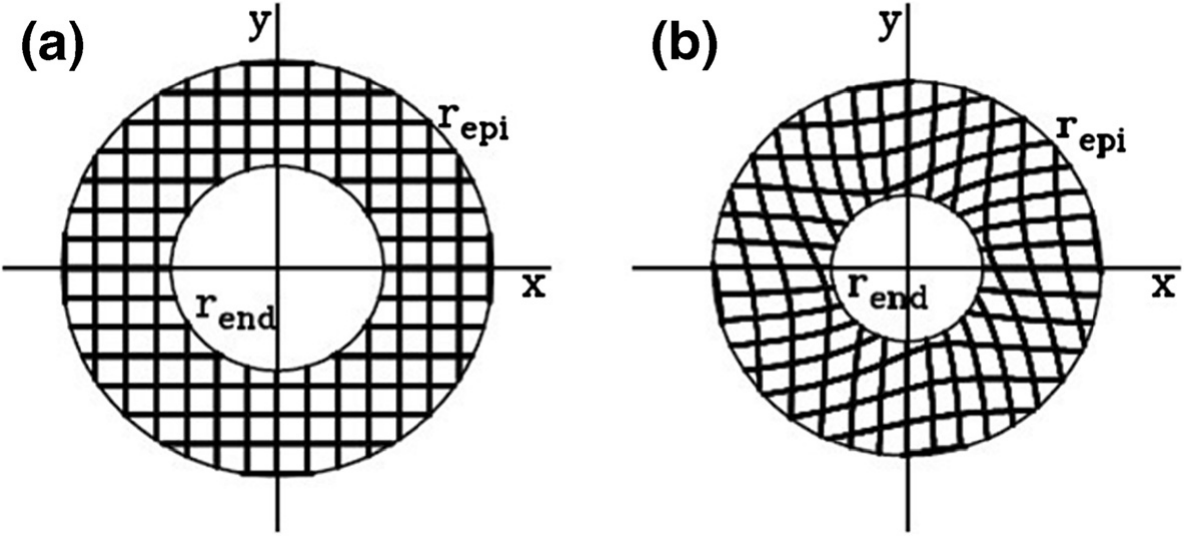
**Example of an annular mesh of simulated tag lines used in model fitting process.** (**a**) Initial uniform mesh. (**b**) Deformed mesh calculated with specific values of the model parameters α and β.

The model was validated by empirically fitting the forward transformation in Eq. (3) successively to the actual motion profile of the LV wall in each image frame with the deformed tagged lines in the series [8]. This process resulted in the estimates of the parameters α and β and their time-dependencies when different frames were concerned. The fitting process started with producing a uniform grid mesh with one pixel line width and also with cell dimensions matched to the tag cells in the initial undeformed image. The simulated mesh was digitally overlaid onto the tagged image as a new layer. The irrelevant tags inside the LV cavity or on the surrounding tissues were excluded by using an annulus. The result was a ring-shaped mesh with uniform grids, as shown in Figure 3a. We then deformed this final mesh by substituting the normalized values α/*r*_*epi*_=0.31 and β/*r*_*epi*_=−0.14, as an example, where the resulting mesh is shown in Figure 3b. The values of α and β were considered optimal if the calculated mesh matched the deformed tag profile in the real LV wall image.

To computationally automatize the fitting process, the tagged image under the consideration at time *t* was adjusted for its contrast and brightness, and then converted to an intensity scaled (gray scale) image *I*_*image*_(*x*^′^,*y*^′^,*t*), Where 0<*I*_*image*_(*x*^′^,*y*^′^,*t*)<1 for all pixels. As above, the new spatial coordinate (*x*^′^,*y*^′^,*t*) was used to denote that the deformation has already been taken place with respect to the undeformed state at (*x*,*y*,*t*=0). The calculated mesh was embedded into an image with intensity distribution *I*_*model*_(*x*^′^,*y*^′^,*α,*β*,t*), such that *I*_*model*_(*x*^′^=*x*,*y*^′^=*y*,0,0,0) was a uniform mesh in Figure 3a overlaid on the undeformed image. After the transformation, *I*_*model*_(*x*^′^,*y*^′^,*α*,β*,t*) assumed zero for all pixels corresponded to the deformed mesh and one otherwise. If the applied transformation were ideal in terms of representing the LV wall motion, this meant that zero intensity pixels in the mesh of *I*_*model*_(*x*^′^,*y*^′^,α,β,*t*) would align perfectly with the dark tag lines of *I*_*image*_(*x*^′^,*y*^′^,*t*) when compared pixel-by-pixel. But, in reality, achieving best alignment required devising a computational approach. In this study, we adapted an optimization strategy where the difference |*I*_*model*_(*x*^′^,*y*^′^,*α,*β*,t*)−*I*_*image*_(*x*^′^,*y*^′^,*t*)| was considered as the cost function and minimized with respect to α and β for every pixel at (*x*^′^,*y*^′^). The overall best match was obtained over the region of the annulus mesh by minimizing a figure-of-merit function

(4) $$F\left(\alpha ,\beta ,t\right)=1/N\Sigma_{i=1:N}{\left[I_{\mathrm{model}}\left(x\prime_{i},y\prime_{i},\alpha ,\beta ,t\right)-I_{\mathrm{image}}\left(x\prime_{i},y\prime_{i},t\right)\right]}^{2}$$

where (*x*^′^_*i*_,*y*^′^_*i*_) and *N* denote the coordinate and number of pixels of the calculated mesh, respectively. Note that *F*(α,β,*t*) ranged from 0 for a perfect match to 1 for a completely mismatch between the calculated mesh and the tags on the LV wall. A minimization of *F*(α,β,*t*) yielded a set of “best-fit” for the parameters α and β. In the model fitting code, the minimization of *F*(α,β,*t*) was accomplished by scanning the two-dimensional α–β space in relatively narrow range as we were only seeking α and β with two or three-digit accuracy.

## Results

**Figure 4:**
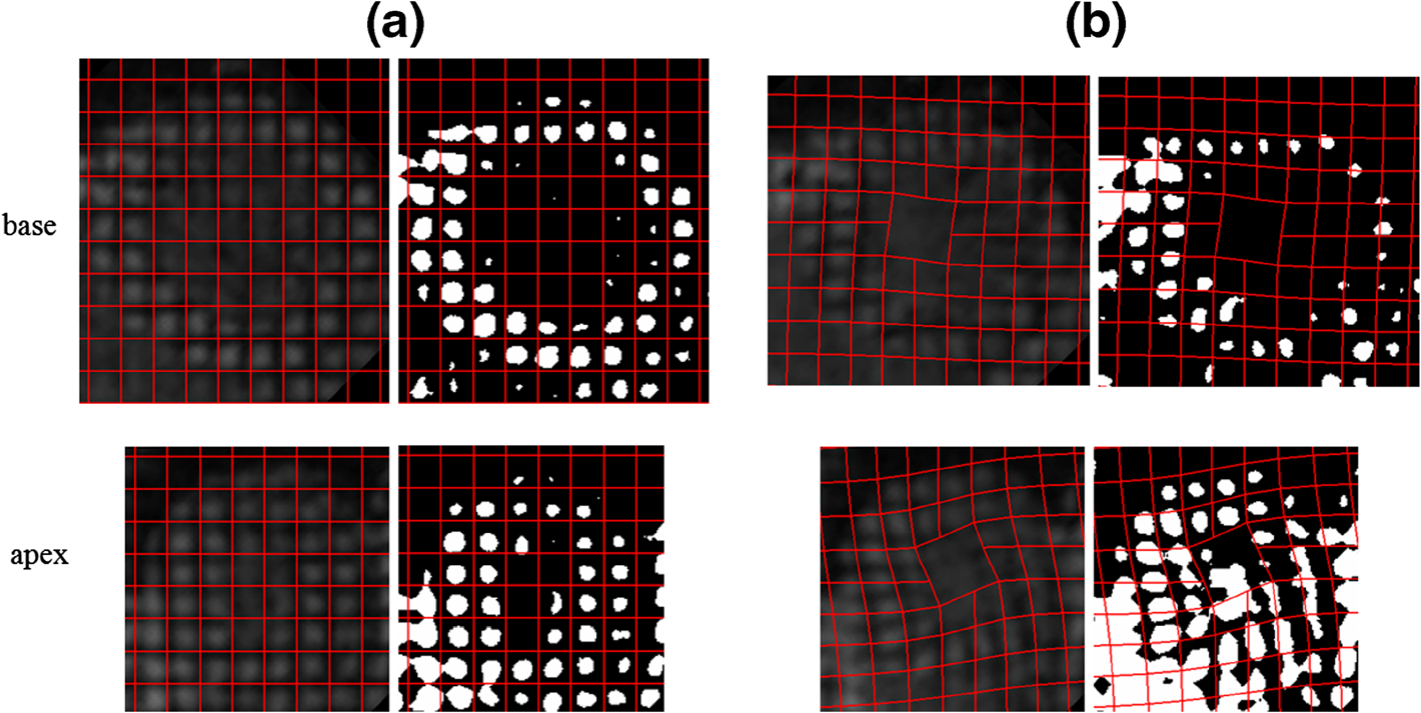
**LV wall at the** (**a**) **beginning and** (**b**) **end of systole as it twists at the base** (**slice** # **9**) **and apex** (**slice** # **4**) **levels in opposite orientations.** The background images on the left are the tagged images of the LV wall and those on the right are the corresponding binary images. The LV wall rotates counter clockwise at the base and clockwise at the apes when viewed from the head-to-toe direction. The meshes of the red lines superimposed on the tagged images are the model predictions of the motions.

In the contiguous tagged images, the 6^th^ slice position corresponded to the mid-ventricle level. The apex and base levels of the LV were identified by the slices 4 and 9, respectively. Figures 4 show examples where the LV wall motions at the apex and base levels were fitted by the model. The superimposed mesh of the red lines were produced by a set of “best-fit” α and β. For an ideal model, the mesh is expected to exactly overlap with the tag grids. Although, the model captured the behavior of the overall motion and followed the tag lines, there were also some discrepancies in between, which indicated the shortcoming of the model. During the minimization processes, the figure-of-merit function in Eq. (4) exhibited reductions that ranged between 15% and 37% and the average reduction was 23% of its maximum.

**Figure 5:**
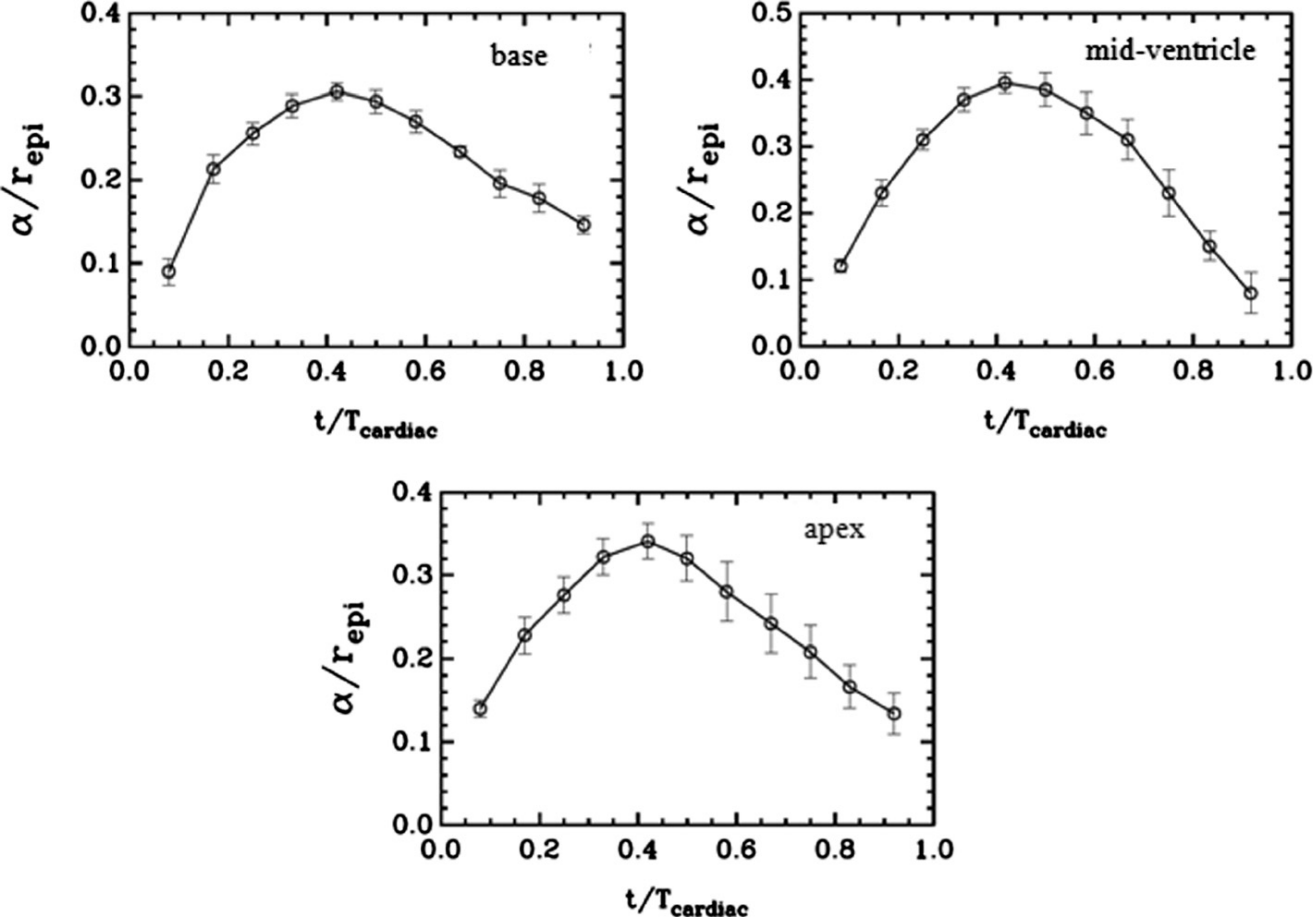
**The variation of the radial displacement parameter** (***α***) **as a function of time within a cardiac cycle at the base** (**slice** # **9**), **mid**-**ventricle** (**slice** # **6**) **and apex** (**slice** # **4**) **levels.***r*_*epi*_ is the end-diastolic radius of the LV wall at the epicardium at the indicated level. *T*_*cardiac*_ is the cardiac period. The error bars represent the intragroup (n=5) standard deviations.

The “best-fit” α values obtained for the LV wall motion at the apex, mid-ventricle and base levels were plotted in Figure 5 as a function of time (*t*) throughout the cardiac cycle. Each error bars on the curves represents the intragroup standard deviation measured at a specific cardiac phase. Note that in the graphs, α was scaled by the radius of the outer wall of the LV wall (epicardium) *r*_*epi*_, and *t* was scaled with the period of the cardiac cycle (*T*_*cardiac*_). These scalings allowed one-to-one comparison of the model-based measurements between the humans irrespective of the differences in the LV wall sizes and cardiac cycle periods. The graphs show that α/*r*_*epi*_ increases during the contraction phase and peaks at about Δ*t*/*T*_*cardiac*_=0.4 then falls off during the relaxation phase of the cardiac cycle at all three levels (apex, mid-ventricle and base).

**Figure 6:**
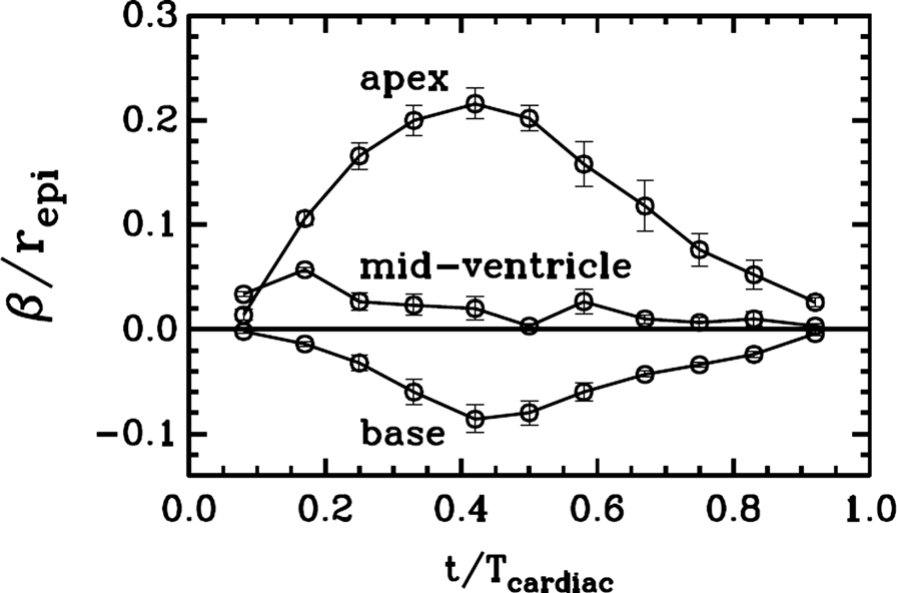
**The variation of the rotation parameter β as a function of time within a cardiac cycle at the base** (**slice** # **9**), **mid**-**ventricle** (**slice** # **6**) **and apex** (**slice** # **4**) **levels.**

The best fit values for the parameter *β* are plotted in Figure 6. For the apex, *β* estimates were positive and attained larger peak (about 0.2) in amplitude as opposed to the negative and lower peak values (about 0.08) at the base level. This was in agreement with the knowledge that the apex twists more than the base.

## Discussion

The use of the tMRI modality was instrumental in building and validating the two-parameter model since the LV wall was conveniently visualized during its contraction and relaxation. Since the tag lines in the acquired images preserved the material coordinates, the lines provided a true system of spatial reference for tracking the motion and matching the simulated grid mesh for determining the model parameters α and β. These parameters could have also been estimated from images acquired with other imaging modalities such as cardiac cine imaging or echography using standard block matching algorithms that work on image intensity features. However, alterations in the intensity pattern of the cine images during the cardiac cycle and speckle decorrelation of the myocardial echogeneity in these modalities would yield estimates with lower precision.

Extensive visual assessments of the images such as those in Figure 4 indicated that the calculated meshes coded by red color mostly overlapped with the tag cells in the LV walls. These qualitative evaluations, combined with the quantitative estimates of the model parameters in Figures 5 and 6, collectively confirmed the validity of the proposed two-parameter model and its capability of representing the underlying LV wall motion reasonably well. The optimization algorithm produced robust and accurate estimates of the parameters α and β at different time points of the cardiac cycle. This might be a generic property of the model that may be extended to cover the LV wall motion in other species. From this aspect alone, the model can potentially be a valuable tool in preclinical translational studies aimed at better understanding and comparing the LV wall motion in experimental studies. The scaling of α and β by *r*_*epi*_ introduced above helps these efforts as it minimizes the intra and inter species variations between the LV wall sizes and the cardiac cycle periods.

Active research focuses on determining specific and sensitive biomarkers at global or regional level for quantitatively evaluating the LV performance under normal or pathological conditions. The two-parameters α and β governing the model can serve this purpose. The model formula in Eq. (3) allows calculating the position of the myocardial tissue in the LV wall at any given spatial point and time in the short-axis plane during the cardiac cycle after the substitution of the α and β estimates promptly. The resulting analytical expressions governing the model can be evaluated to derive additional parameters as surrogate biomarkers for assessing the ongoing cardiac events. For example, from Eq. (1), the ratio α^2^/*r*_*end*_ represents the change in the radius (*r*-*r*^′^) and the reduction of the cross-sectional area of the LV cavity in the short-axis plane is π(*r*^2^-*r*^′^^2^)|_*r*=*end*_. Using the approximation (*r*^2^-*r*^′^^2^) ~ (*r*+*r*^′^) (*r*-*r*^′^) ~ 2*r*(*r*-*r*^′^), α^2^ can be seen as proportional to the reduction of the LV cavity area due to the contraction. The characteristics of such markers could potentially be developed into critical differential diagnostics between the healthy and diseased hearts. One possibility of potential application is in evaluating diabetic heart where the myocardial tissue contraction is modified due to fibrotic tissue deposition. Other possibilities are cases where the heart goes through remodeling after infarct or myocardial hypertrophy. The current preliminary study was, however, performed on a limited number of subjects. By increasing the number of subjects further and performing statistical analysis on the α and β estimates can yield group averages to better embody the mean biomechanics of the LV wall motion as normative measures in healthy individuals. Therefore, evaluations of how well the model would perform in the circumstances of cardiac disease and further extension of the model to represent the overall LV wall motion in every transverse plane remain to future studies.

We note that the model is valid for representing the global cardiac motion only. This presents a limitation since it does not distinguish the differences in regional motions occuring at different segments of the short-axis of the LV wall due to local myocardial abnormalities. Hence, all the regional cardiac function measurements, such as displacement, strain and strain rate, using the model would remain the same for each segment.

Another limitation of the model is that it fails to account for the counter-clockwise rotation of the apex that occurs early on during systole. Insufficient temporal resolution between the image frames may explain such aspect of the apex motion was not present in our data.

Our model fitting procedure involved converting tagged images to binary images. The tag lines typically fade towards the end of the cardiac cycle in the image series. This is a common event and the tag line fading gets worse with a shorter T1 relaxation time of the underlying myocardial tissue or longer period of cardiac cycle. Our study was conducted on a 3 T clinical scanner. T1 measures longer with increased magnetic field, but still this effect was visible in our data. Nevertheless, the model fitting code was able to perform well since the code operated on binary images not the original tagged images. This enabled matching the faded tag lines to the calculated meshes. The ability to perform the fitting process seamlessly using images with relatively poor quality of tag lines is another advantage of our approach.

## Conclusion

This study demonstrated that it is feasible to empirically model the spatial and temporal evolution of the LV wall motion using a two-parameter formulation in conjunction with tMRI-based visualization of the LV wall in the transverse planes at the apex, mid-ventricle and base levels. The algorithm developed and implemented in a simple fashion provides robust and accurate estimates of the two-parameters of the model when tMRI data was from normal human hearts. The performance of the estimator does not degrade with the fading of the tag lines. Once a model is constructed, a measure of interest characterizing certain aspect of the LV wall biomechanics, such as strain, strain rate or torsion, can be derived and expressed analytically. Any deviation in such measure from its normative value would indicate cardiac abnormality, and thereby serve as a surrogate biomarker for detecting cardiac dysfunction and evaluating its severity in clinical investigations or experimental studies with translational focus.

### Appendix

The inverse transformation regarding Eq. (3) can be written as

(5) $$r=\left(RT\right){-}^{1}r'$$

or

(6) $$\left[\begin{array}{c} x\\ y \end{array}\right]=\left[\begin{array}{c} \frac{1}{2}\left(1+\sqrt{1+4\frac{{\text{α}}^{2}\left(t\right)}{r{'}^{2}}}\right)\left\{x'\cos\left(\frac{2\beta \left(t\right)}{r'+\sqrt{r{'}^{2}+4{\text{α}}^{2}\left(t\right)}}\right)+y'\sin\left(\frac{2\beta \left(t\right)}{r'+\sqrt{r{'}^{2}+4{\text{α}}^{2}\left(t\right)}}\right)\right\}\\ \frac{1}{2}\left(1+\sqrt{1+4\frac{{\text{α}}^{2}\left(t\right)}{r{'}^{2}}}\right)\left\{-x'\cos\left(\frac{2\beta \left(t\right)}{r'+\sqrt{r{'}^{2}+4{\text{α}}^{2}\left(t\right)}}\right)+y'\sin\left(\frac{2\beta \left(t\right)}{r'+\sqrt{r{'}^{2}+4{\text{α}}^{2}\left(t\right)}}\right)\right\} \end{array}\right]$$

where *r* ^′^ = (*x* ^′^ ^2^ + *y* ^′^ ^2^)^1/2^. When the radial displacement is small (4*α*^2^/*r* ' ^2^ < < 1), the approximation (1 + √ (1 + 4*α*^2^/*r* ' ^2^))/2 ≃ 1 + *α*^2^/*r* ' ^2^ holds. But, the radial displacement remains large during most cardiac cycle especially near the end systole and 4*α*^2^/*r* ' ^2^ ∼ 1 holds.

## Competing interests

The authors declare that they have no competing interests.

## Authors’ contribution

JJS contributed to the development of the analytical model presented in the paper and fitting and interpretation of the numerical data. MA performed the numerical analysis. IVS contributed to the study plan and experimental data collection and evaluation. MB conceived the initial study and helped with the collection of cardiac imaging data, interpretation of the overall numerical and experimental results, preparation of the manuscript and its revision. All authors read and approved the final manuscript.
